# Physical exercise habits are related with reduced prevalence of falling among elderly women in China

**DOI:** 10.1186/s12905-023-02808-z

**Published:** 2023-12-08

**Authors:** Cuiqing Zhao, Tongling Wang, Dawei Yu, Wang Li

**Affiliations:** 1 Division of Physical Education, Myongji University, Seoul, 03674 South Korea; 2 Institute of Physical Education, Huzhou University, Huzhou, Zhejiang Province 313000 People’s Republic of China; 3 Department of Physical Education, Huaiyin Institute of Technology, Huaian, Jiangsu Province 223003 People’s Republic of China

**Keywords:** Accidental falls, Aged female, Cross-sectional study, Exercise habits

## Abstract

**Background:**

Although some studies have examined the association between exercise and falls, most have focused on specific exercises, and the results have been inconsistent. In addition, there is a lack of evidence on elderly Chinese women who have different living and exercise habits compared to those in other countries. Therefore, this study aimed to investigate whether physical exercise is associated with falls in elderly Chinese women.

**Methods:**

This cross-sectional study included 1429 elderly Chinese women with a mean age of 69.2 years. Information on physical exercise habits and fall experiences was collected using a self-report questionnaire. Logistic regression models were used to analyze the association between physical exercise habits and falls.

**Results:**

The results showed that 15% participants had a fall in the past year. After adjusting for confounding factors, the odd ratios (ORs) and 95% Confidence Intervals (CIs) for fall experiences across categories of exercise frequency were as follow: 1 (reference) for no exercise behavior, 0.50 (0.29, 0.85) for exercise 1 to 5 times a week, and 0.37 (0.25, 0.55) for exercise more than 6 times a week. Furthermore, the ORs (95% CIs) across categories of exercise insistence were 1 (reference) for less than 1 year, 0.78 (0.37, 1.65) for 1 to 3 years, and 0.38 (0.20, 0.74) for more than 3 years. In terms of exercise duration, the ORs (95% CIs) for < 1 h/day, 1–2 h/day, and > 2 h/day were 1 (reference), 0.85 (0.53, 1.36), and 2.80 (1.30, 6.05). Unlike other variables, longer exercise duration was associated unfavorably with falls.

**Conclusion:**

Physical exercise habits were associated with falls in elderly Chinese women. Keeping a proper exercise habit may contribute to lower risk of falling in elderly women.

## Background

China has become an aging society with more than 18% of its population being above 60 years old. Among them, 14.2% are 65 years or older [1]. By 2050, the country will be a super-aged society because the elderly is forecasted to constitute more than 30% of the total population [2]. One’s physical and mental abilities generally decline with age [3, 4]. For the elderly, the prevalence of falling increases with the degeneration of their physical functions and worsening of their mental and cognitive health. Globally, approximately 28–35% of the elderly above the age of 65 suffer from falls annually. For those over the age of 70, the rate of falling increases to 32–42%. [5]。Studies have shown that 31% of those who fell suffered external trauma (such as fractures) that required medical attention, which led to limited mobility and in turn, more severe consequences [6]. The number of falls and fractures is likely to increase with the growing elderly population in China, which will be accompanied by external trauma caused by falling and the early occurrence of the fears of falling and being bedridden. The associated social problems, such as increasing medical expenses, are also expected to worsen in the future [7].

The causes of falls have been studied and are categorized into internal and external. The internal causes include physical and psychological factors; the external causes pertain to the environment, such as uneven terrain and slippery grounds [8]. In contrast to external causes becoming less significant with the aging of a person, internal causes increase in their significance [9]. Correspondingly, effective measures can be arrived at by focusing on those internal causes. Randomized controlled trials have indicated that training which improves balance and enhances lower body strength has a preventative effect on falls [10, 11]. During randomized controlled trials, the impacts of confounding factors can be controlled and information such as experimental participants and contents can be properly managed. Nevertheless, such studies were completed under the guidance of professionals, conducted in special environments (such as specialized scientific research or medical institutions), and generally lasted a relatively short period of 3–6 months. These conditions differ substantially from real life environments. Moreover, most people do not have the opportunity of receiving proper instructions on exercising. There are also significant variations between individuals in terms of exercise duration, frequency, and intensity. In addition, some of these studies have small sample size and the majority primarily assess the risk of falls indirectly through measures of physical capability rather than actual fall experiences. On the other hand, some studies have highlighted that certain types and patterns of physical activity might increase the risk for falls and fractures [12]; exercise intensity and volume have also been shown to be associated with falls [13, 14]. Following from the above, it appears that there was a lack of complete consistency among the conclusions arrived at by previous studies. Thus, more research is required to gather evidence for proving the relationship between physical exercise and the prevalence of falling.

It was noted that the living habits of elderly females in China may differ from those in other countries; they are also more willing to participate in group-based physical activities that are culturally unique. Therefore, it was necessary to verify whether exercise habits were associated with falls for this population. Previous studies concluded that appropriate exercises had positive impacts on physical functions, which implied a reduction in the risks of falling. However, there has not been any large-scale survey involving a huge population to verify the relationship between exercise and actual fall experiences among elderly females in China. Hence, a cross-sectional study was designed to determine whether such a correlation existed, as well as whether there was any correlation between various exercise components including exercise frequency, duration, intensity, and the period over which the exercise habits were maintained and the prevalence of falling in elderly Chinese women.

## Methods

### Participants

The participants of this study were elderly females above the age of 60 years old who had previously attended a bone health examination for local residents, which was conducted for elderly females at the Jiuhua Health Management Center in the Fengxian District of Shanghai from April to May 2019. A face-to-face survey was performed after the health examination in a separate room. All participants who had undergone the examination were then invited to voluntarily participate in our study. Those who agreed provided their written consent after receiving an explanation of the purpose of the study. Subsequently, they underwent a health check before we distributed our questionnaires to them. The present study was conducted in accordance with the Declaration of Helsinki, and the protocol was approved by the Ethics Committee of the Huaiyin Institute of Technology.

$$N = \frac{{{{\left[ {{Z_\alpha }\sqrt {2\bar p(1 - \bar p)} + {Z_\beta }\sqrt {{p_1}(1 - {p_1}) + {p_2}(1 - {p_2})} } \right]}^2}}}{{{{({p_1} - {p_2})}^2}}}$$

**Fig. 1:**
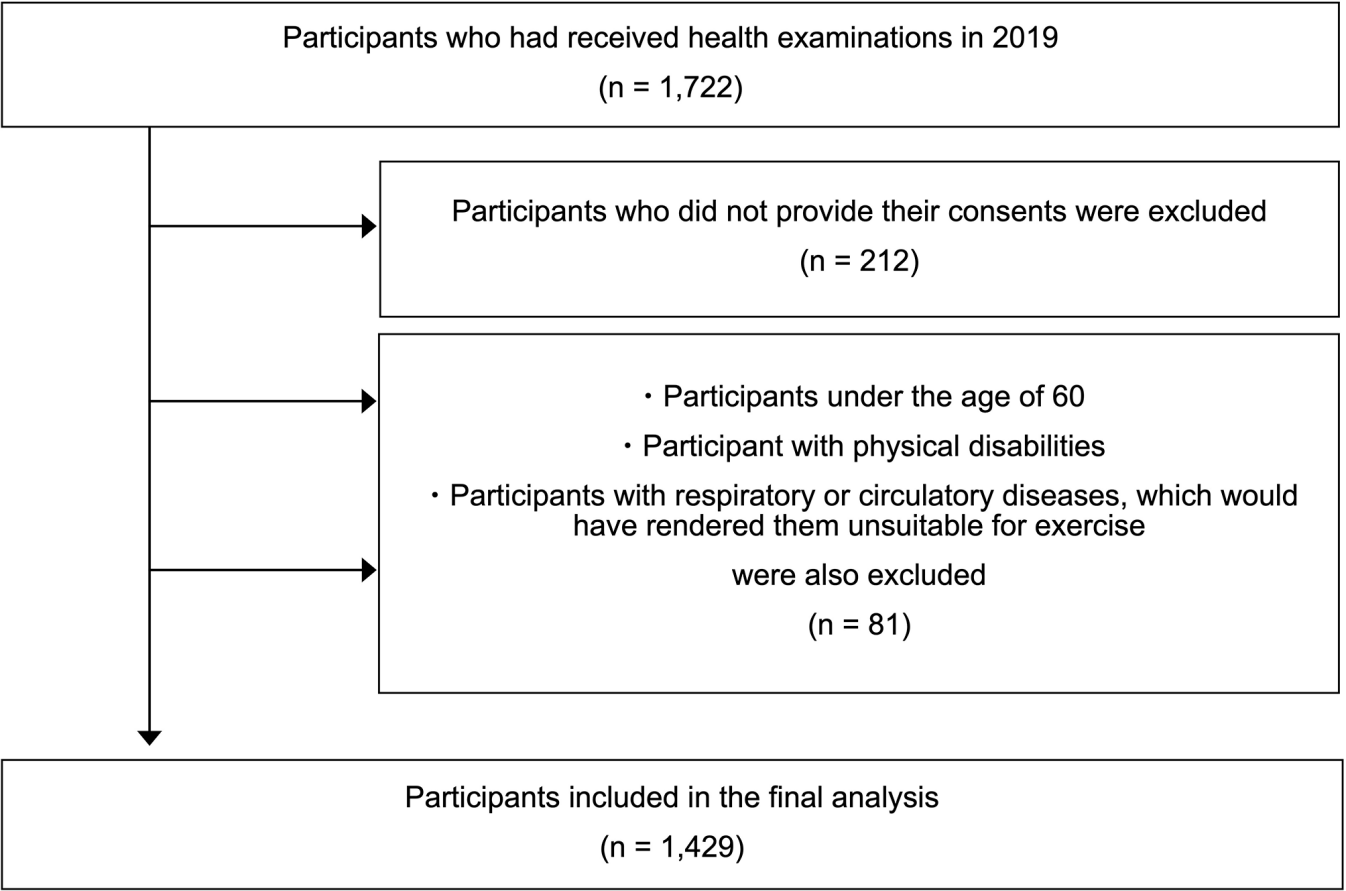
Flow chart of the sample selection process

The exclusion criteria for this study were: (i) persons under the age of 60; (ii) persons with physical disabilities; and (iii) persons with respiratory or circulatory diseases, which would have rendered them unsuitable for exercise. A total of 1,510 persons agreed to participate in the study. After eliminating those persons with missing values or were outliers in their questionnaire or health check data (*n* = 81), the final sample size for the study was 1,429. (Fig. 1) Study ample size was calculated using the following formula, and our sample size is sufficient for assessing the association of exercise frequency and other exercise behaviors with experience of falls.

### Assessment of physical exercise

Physical exercise was assessed using a self-reported questionnaire which has been used in previous study [15], that included four components as follows: (1) Exercise frequency was assessed by the question, “During the last month, how many days did you exercise per week?” Responses ranged from never to seven days. Responses were divided into three categories: “Never,” “1 day to 5 days,” and “≥ 6 days.” (2) Intensity of exercise was assessed by the question, “What is the usual intensity of your exercise?” The response options were: “Low intensity: breathing and heart rate does not change significantly, and the exercise is not challenging to perform”; “Intermediate intensity: slightly faster breathing and heart rate with minimal sweating”; and “High intensity: rapid breathing, faster heart rate, and profuse sweating.” (3) Duration of exercise was assessed by the question, “How long do you usually exercise?” The possible responses were “< 30 minutes,” “30 minutes to 1 hour,” “1 to 2 hours,” and “> 2 hours.” Responses were then divided into three categories: “< 1 hour,” “1 to 2 hours,” and “> 2 hours” for analysis. (4) History of exercise was assessed by the question, “How long have you been exercising?” Possible responses were “< 6 months,” “6 months to 1 year,” “1 to 3 years,” “3 to 5 years,” and “> 5 years.” For the analysis, we divided the responses into three categories: < 1, 1–3, and > 3 years. The test score reliability coefficient was 0.95 using Cronbach’s alpha.

### Assessment of fall experiences

Self-reported fall experiences were evaluated by asking, “Did you have any falls in the past year?” The possible responses were “Yes” or “No”. Participants who responded “Yes” were asked to indicate the number of times they fell. Because there were few participants with more than one fall experience, we did not perform any subgroup analyses.

### Covariates

The participants’ body mass index (BMI) was calculated using the participants’ weight and height data, which were measured during their health examinations. In addition, an automatic sphygmomanometer (KENTARO HBP-9021 J, Japan) was used to measure their blood pressure data. Those meeting one of the following conditions were judged to have hypertension: (1) Systolic blood pressure ≥ 140 mmHg; (2) diastolic blood pressure ≥ 90 mmHg; or (3) use of antihypertensive drugs [16]. Depressive symptoms were assessed using the Self-Rating Depression Scale (SDS) [17]. The threshold for depressive symptoms was 40 points on the SDS [18]. Sociodemographic information was obtained using a self-reported questionnaire, including age (continuous variable), former occupation (white or blue collar), tobacco smoking status (smoker or non-smoker), alcohol drinking status (everyday, occasionally, or non-drinker), household income (low income, ≤ 50,000 Yuan; middle income, 50,001–70,000 Yuan; or high income, > 70,000 Yuan), living conditions (living alone or not), and educational level (< high school diploma or ≥ high school).

### Statistical analyses

The *t*-test and chi-square test were used to compare group differences in participants’ characteristics according to their fall experiences. Categorical and continuous variables were expressed as mean (95% confidence intervals (CIs)) and n (%), respectively. Logistic regression analysis was performed to estimate the crude odds ratios (ORs) and CIs for the association between physical exercise and fall experience. Fall experience was used as the dependent variable, and physical exercise habits were used as independent variables. Multiple logistic regression analysis was performed to adjust for confounders. We analyzed both Model 1 (adjusted for age and BMI) and Model 2 (adjusted for the factors in Model 1 plus household income, former occupation, living condition, educational level, tobacco smoking and alcohol drinking status, hypertension, diabetes, and depression). All statistical analyses were performed using the Statistical Package for Social Science software (version 24.0; SPSS, Inc., Chicago, IL, USA). Statistical significance was set at *p* < 0.05.

## Results

Table 1 shows the basic characteristics of participants with and without a fall experience in the past year. 15% of all participants and 13% of participants with exercise habits experienced falls in the past year. In all participants, those of younger age, higher BMI, smoking habits, higher occasional alcohol consumption, and diabetes tended to have a fall episode. On the other hand, in participants with exercise habits, those of younger age, higher occasional alcohol consumption, diabetes, and those who did not live alone tended to have fall experiences.

**Table 1:** Participant characteristics according to fall experiences within the past year

	All participants		*p* value^1^	Participants with exercise habits		*p* value^1^
	*n* = 1429			*n* = 833		
	Fall experience			Fall experience		
	No	Yes		No	Yes	
*n* (%)	1215 (85.0)	214 (15.0)		725 (87.0)	108 (13.0)	
Age	69.6 (69.2, 70.0)^2^	66.7 (65.7, 67.6)	< 0.001	68.7 (68.3, 69.2)	63.9 (62.7, 65.1)	< 0.001
BMI	24.1 (24.0, 24.3)	24.8 (24.4, 25.2)	0.002	24.5 (24.3, 24.6)	24.2 (23.7, 24.7)	0.359
Former occupation (*n*; %)						
White color	202 (16.6)	34 (15.9)	0.842	154 (21.2)	26 (24.1)	0.531
Blue color	1013 (83.4)	180 (84.1)		571 (78.8)	82 (75.9)	
Smoking (*n*; %)						
Smoker	31 (2.6)	0 (0)	0.010	25 (3.4)	0 (0)	0.063
Non-smoker	1184 (97.4)	214 (100)		700 (96.6)	108 (100)	
Alcohol drinking (*n*; %)						
Drinking everyday	16 (1.3)	0 (0)	< 0.001	13 (1.8)	0 (0)	< 0.001
Drinking occasionally	31 (2.6)	40 (18.7)		22 (3.0)	32 (29.6)	
Non-drinker	1168 (96.1)	174 (81.3)		690 (95.2)	76 (70.4)	
Household Income (*n*; %)						
Low	448 (36.9)	68 (31.8)	0.165	182 (25.1)	24 (22.2)	0.116
Middle	367 (30.2)	70 (32.7)		253 (34.9)	32 (29.6)	
High	400 (32.9)	76 (35.5)		290 (40.0)	52 (48.1)	
Living alone (*n*; %)						
Yes	104 (8.6)	12 (5.6)	0.174	32 (4.4)	0 (0)	0.016
No	1111 (91.4)	202 (94.4)		693 (95.6)	108 (100)	
Educational level (*n*; %)						
≥ High school	264 (21.7)	46 (21.5)	1.000	112 (15.4)	14 (13.0)	0.567
< High school	951 (78.3)	168 (78.5)		613 (84.6)	94 (87.0)	
Hypertension (*n*; %)						
Yes	718 (59.1)	120 (56.1)	0.409	422 (58.2)	62 (57.4)	0.917
No	497 (40.9)	94 (43.9)		303 (41.8)	46 (42.6)	
Diabetes (*n*; %)						
Yes	198 (16.3)	58 (27.1)	< 0.001	86 (11.9)	34 (31.5)	< 0.001
No	1017 (83.7)	156 (72.9)		639 (88.1)	74 (68.5)	
Depression (*n*; %)						
Yes	511 (42.1)	89 (41.6)	0.940	249 (34.3)	36 (33.3)	0.914
No	704 (57.9)	125 (58.4)		476 (65.7)	72 (66.7)	

^1^ Obtained using t-test for continuous variables and chi-square analysis for variables of proportion

^2^ Mean (95% CI) for all continuous variables

Table 2 shows the association between exercise frequency and fall experiences in the past year among all participants (1429). Compared with the no-exercise group (reference), the ORs (95% CIs) of 1–5 and ≥ 6 days/week of exercise in the crude model were 0.61 (0.38, 0.98) and 0.72 (0.52, 0.98), respectively (*p* for trend = 0.033). In the final adjusted model (model 2), this inverse association was strengthened. In addition, compared with the no-exercise group (refence), the ORs (95% CIs) of 1–5 and ≥ 6 days/week of exercise were 0.50 (0.29, 0.85) and 0.37 (0.25, 0.55), respectively (*p* for trend < 0.001).

**Table 2:** Adjusted associations between exercise frequency and fall experiences in the past year among 1,429 older women

	Exercise frequency (days/week)			*p* for trend^1^
	0	1–5	≥ 6	
*n*	596	206	627	
With fall experience	106	24	84	
Prevalence (%)^2^	17.8	11.7	13.4	
Crude model	1	0.61 (0.38, 0.98)3^*^	0.72 (0.52, 0.98)^*^	0.033
Model 1^4^	1	0.53 (0.33, 0.86)^*^	0.58 (0.42, 0.80)^*^	0.001
Model 2 ^5^	1	0.50 (0.29, 0.85)^*^	0.37 (0.25, 0.55)^*^	< 0.001

^1^ Obtained by multivariate logistic regression analysis

^2^ Prevalence of falls based on the number of fall experiences in each tertile

^3^ Values represent odds ratios and 95% confidence intervals

^4^ Adjusted for age and BMI

^5^ Adjusted for age, BMI, former occupation, educational level, living status, family income, hypertension, diabetes, and smoking and drinking habits

^*^ Significantly different from the reference category (*p* < 0.05)

The associations among exercise duration, intensity, and incidents of falls in the past year are shown in Table 3. In the final adjusted model of exercise duration, the OR (95% CI) of long exercise durations (> 2 h) was 2.80 (1.30, 6.05), which was significantly higher than those of the short (< 1 h) and medium (1–2 h) exercise durations. However, no linear association was observed. Additionally, no significant association was found between exercise intensity and fall experiences in the crude and adjusted models. Compared with < 1 years of exercise (reference), the ORs (95% CIs) of 1–3 and > 3 years in the crude model were 0.55 (0.30, 1.01) and 0.43 (0.24, 0.77), respectively (*p* for trend = 0.007). This significant inverse association did not change in Models 1 and 2 (*p* for trend = 0.001 for both models).

**Table 3:** Adjusted associations between exercise behaviors and fall experiences in the past year among 833 older women

	Categories of exercise behaviors			
	Exercise duration (hours/day)			*p* for trend^1^
	< 1	1–2	> 2	
*n*	374	391	68	
With fall experience	52	44	20	
Prevalence (%)^2^	13.9	11.3	29.4	
Crude model	1	0.79 (0.51, 1.21)^3^	2.58 (1.42, 4.69)^*^	0.092
Model 1^4^	1	0.85 (0.55, 1.32)	2.72 (1.46, 5.07)^*^	0.054
Model 2^5^	1	0.85 (0.53, 1.36)	2.80 (1.30, 6.05)^*^	0.174
	Exercise intensity (level)			
	Low	Middle	High	
*n*	188	537	108	
With fall experience	20	74	16	
Prevalence (%)	10.6	13.8	14.8	
Crude	1	1.34 (0.80, 2.27)	1.46 (0.72, 2.96)	0.254
Model 1^4^	1	0.94 (0.53, 1.65)	0.91 (0.41, 2.01)	0.807
Model 2^5^	1	0.84 (0.47, 1.49)	0.86 (0.37, 1.97)	0.655
	Exercise history (year)			
	< 1	1–3	> 3	
*n*	90	252	491	
With fall experience	20	34	54	
Prevalence (%)	22.2	13.5	11.0	
Crude model	1	0.55 (0.30, 1.01)	0.43 (0.24, 0.77) ^*^	0.007
Model 1^4^	1	0.75 (0.39, 1.43)	0.40 (0.22, 0.73)^*^	0.001
Model 2^5^	1	0.78 (0.37, 1.65)	0.38 (0.20, 0.74)^*^	0.001

^1^ Obtained by multivariate logistic regression analysis

^2^ Prevalence of falls based on the number of fall experiences in each tertile

^3^ Values represent odds ratios and 95% confidence intervals

^4^ Adjusted for age and BMI

^5^ Adjusted for age, BMI, former occupation, educational level, living status, family income, hypertension, diabetes, and smoking and drinking habits

^*^ Significantly different from the reference category (*p* < 0.05)

## Discussion

In this study, the association between physical exercise habits and fall experiences in the past year was examined in elderly Chinese women. The findings indicate that those with high exercise frequencies, and a long exercise history had a decreased odds of experiencing falls. On the other hand, longer exercise duration was associated with a higher odds of falling. These associations were independent of confounding factors. However, exercise intensity was not associated with falls. To our knowledge, this is the first study to suggest that exercise habits may be effective in preventing falls among elderly Chinese women.

In the literature, a high proportion of fall experiences have been reported. For example, a New Zealand study on 71,856 elderly aged over 65 years showed that 40.8% experienced falls during past 90 days [19]. Another study in 606 Ethiopian community-dwelling older adults aged 50 and over, reported that 28.4% of participant fell at least once in the within a one-year period [20]. In contrast, the fall rates in some Asian studies were consistent with our study. For example, the incidence of falls in a 1-year period was reported to be 16.4% in older Chinese people aged 60–95 living in Hong Kong [21]. Yang et al. reported a 15.1% fall rate in 1,067 older adults aged 65 years and over in Taiwan [22]. In addition, a study on 430 middle-aged and older Japanese individuals indicated that 15.6% of study subjects had experienced a fall in the past year [23]. Furthermore, a Chinese study reported a 11.9% fall rate in 5,374 older adults over 60 years old [24].

Although there is no direct evidence in the literature regarding the association between physical exercise and falls in the elderly Chinese population, the findings of some previous studies from other countries may support our study. For instance, Roller et al. indicated that modified Pilates exercises performed once a week over 10 weeks reduced the fall risk in adults aged 65 and older [25]. Antonino et al. also reported that 13-week Pilates exercise could be considered to reduce the risk of falls in the elderly [26]. However, in their study, the sample size was too small, and the percentage of male participants was unbalanced in each group, that may have led to selection bias. In another study, Sousa et al. indicated that 32 weeks of combined resistance and aerobic exercise and aerobic exercise alone were more effective than no exercise in reducing fall risk among men aged 65–74 [27]. But the participants in this study were only men, the relationship in women was not evaluated. Moreover, Sungkarat et al. reported that in older adults aged 61–75, the performance of Tai Chi three time a week for 15 weeks had great effects on reducing the risk of falls [28]. The number of men in this study was quite limited, and they were unevenly distributed between control and Tai Chi group (7 vs. 2). This could potentially influence the results, as the impact of exercise may differ between genders. Summarizing these studies, they all include balance training. Additionally, two other studies have also shown that balance training can decreased the risk of falls in older people [29, 30]. Overall, although the types of exercises differed, all of these previous studies showed that physical exercise was effective in reducing the risk of falls in older adults. However, these studies exhibit certain limitations. Such as, they mostly focus on the elderly population as a whole or on men, with little attention to women or gender differences. Moreover, these studies assessed falls using fitness tests such as the chair stand test, one-leg stance test, and Timed Up and Go test, as opposed to real-life fall experiences. These tests were used to assess participants’ balance, which may be a component in the evaluation of one’s risk of falling; however, these tests cannot represent falls directly. Additionally, there is no epidemiological evidence regarding these associations in the elderly Chinese population. Therefore, this study fills this gap in elderly Chinese women. On the other hand, we found that long exercise duration is associated with a high odd of falls in present study. This is consistent with a review study that indicates that some activities, particularly those of high intensity or long duration may put women at an increased risk of falls [12]. This causes concern that physical activity may increase the risk of falls in older people [31]. Thus, the result of our study strengthened evidence on the field of physical activity on elderly, and suggests that older adults should avoid prolonged physical activity, as falls can lead to fractures [32], which, for older individuals, can result in more serious outcomes.

In contrast to the findings above, one study reported results that were inconsistent with our findings. The study explored the associations between various types of activities and falling in community-dwelling older persons, reporting that performing household activities was associated with a decreased risk of falling and that performing sports or activities with high intensity was associated with an increased risk of falling [13]. However, as we did not collect any information on household activities in our study and we are unable to make a direct comparison to our results in this regard. Notably, the participants in our study who had physical exercise habits had a lower odd of falls, and exercise intensity was not found to be associated with fall experiences, which differs from the results of the aforementioned study. Several differences between these studies may explain these contrasting findings. First, different measures of falls and physical exercise were assessed. Second, the participants were from different areas. Our study was conducted in Shanghai, a relatively warm city, in winter. In contrast, the other study was conducted in Amsterdam, which has a long winter; therefore, people are more prone to slip if they exercise outside, which may lead to a higher fall rate in people who exercise.

The mechanism by which physical exercise leads to a low prevalence of falls can be explained as follows. First, it has been reported that physical exercise is effective in improving muscle strength and balance [33, 34]. Strong muscle strength and a high ability to maintain balance have been associated with a decreased risk of falling [22, 35]. In addition, it has also been indicated that physical exercise is associated with a lower occurrence of obesity. Notably, older individuals with higher body weights have an increased risk of falls [36]. Therefore, physical exercise could be related to a lower prevalence of falls.

Our study has several limitations that must be considered. First, because fall experiences were assessed by self-reporting, a recall bias may exist. Second, data on confounding factors were limited, which means that there may be other factors that influence the association between physical exercise and fall experiences, such as dietary and medication information. Third, because of the cross-sectional nature of the study, causality cannot be concluded. Fourth, the participants in this study were exclusively recruited from a bone health examination program in Shanghai. Thus, a selection bias exists, and the present study sample may not represent all elderly women. Further studies are required to determine whether these associations can be replicated in other populations. Finally, due to the limited survey time and space on the questionnaire, we assessed only fall experiences within the past year in present study. Other aspects of falls, such as the location of falls, contributing factors, or the severity of the falls, were not included. Consequently, our discussion of falls in this study may not be comprehensive. These factors should be considered in the future studies.

## Conclusion

The present study is the first to provide evidence of the association between physical exercise elements and fall experiences in elderly Chinese women. The results suggest that a higher frequency of exercise and a long exercise history may contribute to a decreased odd of falls. In contrast, exercise durations greater than two hours may be a risk factor for falls in elderly women. Our finding strengthens the evidence on the association between physical exercise and fall, and highlight the importance of physical exercise in elderly population, further provides important evidence for women’s physical health and implications for the prevention of falls and fractures. However, there is a need to examine causality using prospective and interventional studies in elderly Chinese women.

## Data Availability

The datasets used and analyzed during the current study are available from the corresponding author on reasonable request.

## List of Abbreviations

BMI: Body mass index
SDS: Zung self-rating depression scale
CI: Confidence interval
OR: Odd ratio
SPSS: Statistical Package for Social Science software
